# Guided and unguided CBT for social anxiety disorder and/or panic disorder via the Internet and a smartphone application: study protocol for a randomised controlled trial

**DOI:** 10.1186/1745-6215-14-437

**Published:** 2013-12-18

**Authors:** Philip Lindner, Ekaterina Ivanova, Kien Hoa Ly, Gerhard Andersson, Per Carlbring

**Affiliations:** 1 Department of Clinical Neuroscience, Karolinska Institutet, Stockholm, Sweden; 2 Department of Psychology, Stockholm University, 106 91 Stockholm, Sweden; 3 Department of Behavioural Sciences and Learning, Linköping University, Linköping, Sweden

**Keywords:** Smartphone, Application, App, Cognitive behavioural, Internet-administered, Anxiety, Randomised controlled trial

## Abstract

**Background:**

Smartphone technology presents a novel and promising opportunity to extend the reach of psychotherapeutic interventions by moving selected parts of the therapy into the real-life situations causing distress. This randomised controlled trial will investigate the effects of a transdiagnostic, Internet-administered cognitive behavioural (iCBT) self-help program for anxiety, supplemented with a smartphone application. The effect of added therapist support will also be studied.

**Methods/Design:**

One hundred and fifty participants meeting diagnostic criteria for social anxiety disorder and/or panic disorder will be evenly randomised to either one of three study groups: 1, smartphone-supplemented iCBT with therapist support; 2, smartphone-supplemented iCBT without therapist support; or 3, an active waiting list control group with delayed treatment. Primary outcome measure will be the Generalised Anxiety Disorder 7-item self-rating scale. Secondary measures include other anxiety, depression and quality of life measures. In addition to pre- and post-treatment measurements, the study includes two mid-treatment (days 24 and 48) and two follow-up assessments (12 and 36 months) to assess rapid and long-term effects.

**Discussion:**

To our knowledge, this is the first study to investigate the effectiveness of smartphone-supplemented iCBT for anxiety disorders. Hence, the findings from this trial will constitute great advancements in the burgeoning and promising field of smartphone-administered psychological interventions. Limitations are discussed.

**Trial registration:**

Clinicaltrials.gov: NCT01963806

## Background

Research has consistently supported the effectiveness of cognitive behavioural self-help programs administered via the Internet (iCBT) [1] for treating depression [2], a variety of anxiety disorders [3] and many conditions within the behavioural medicine field, for example, tinnitus [4] and irritable bowel syndrome [5]. The addition of a therapist to guide the patient through the self-help program has been found to increase effect sizes [6,7] to near-equal or even equal to those of traditional, face-to-face cognitive behavioural therapy (CBT) [8,9]. Guided iCBT has demonstrated both efficacy and effectiveness [10], with similar effect sizes seen when implemented in routine psychiatric care, for example, in treatment of panic disorder [11,12]. Higher cost-effectiveness than group psychotherapy has also been demonstrated (for example, [13]).

Despite their proven effectiveness, current iCBT programs leave room for improvement. Just as in traditional face-to-face CBT, psychotherapeutic information (psychoeducation, teaching of skills, task assignment and more) is conveyed to the patient (in iCBT, often in the form of reading modules), who then carries out the assigned tasks and afterwards records or reports progress. In essence, in current iCBT, the computer replaces the therapy room yet the overall format remains the same. Hence, just as in traditional CBT [14], helping the patient translate and implement what has been learned in the safe therapeutic environment into real-life remains a challenge.

The increasing everyday usage of smartphones presents an exciting and promising opportunity to extend the reach of psychological interventions and thereby their effectiveness [15]. Smartphone technology, in the form of tailored applications (apps) may be used as supplementary iCBT components allowing novel features such as in-context access to psychoeducational material and automated, tailored messages, reminders and feedback, as well as live reporting of behaviours, thoughts and feelings unbiased by retrospective recall. Smartphone technology thus enables the therapist to move selected parts of the therapy outside of the session and into the real-life situations associated with distress or impairment.

The relatively few studies conducted so far indicate that smartphone-administered interventions are indeed effective. A recent randomised controlled trial (RCT) contrasting smartphone- *vs*. computer-delivered CBT-style self-help for depression found equal improvements in the two groups [16]. Encouraging initial results have also been found in mobile phone-based behavioural interventions aimed at smoking cessation [17], increasing physical activity [18], among others. Preliminary research also supports using smartphone applications to collect valid psychiatric data [19]. Although the initial results are promising, the question of how to best make effective use of smartphone applications for improving mental health remains unanswered. Importantly, we are not aware of any study so far targeting anxiety disorders. Whether the addition of therapist support increases effects, as seen in regular iCBT [6,7], also remains to be investigated. iCBT smartphone applications are able to give immediate, automated feedback, which could prove as effective as personal feedback from a therapist. Further, since these programs are often designed to be more user-friendly than regular iCBT programs, low treatment compliance - which is associated with worst treatment outcomes - may be a lesser issue, which means there may be less need for a therapist to encourage compliance. If the results of this trial indicate equal effects regardless of whether therapist support is added or not, this will have a great impact on how future smartphone-supplemented iCBT research is designed. Until equal effects has been demonstrated, prior research suggests great treatment effects with added therapist support.

The purpose of the RCT described in this study protocol is two-fold: first, to investigate the effectiveness of a transdiagnostic iCBT program supplemented with a smartphone application for two common [20,21] anxiety disorders; and second, to directly compare two ways of delivering this program, either with or without added support by a therapist. Both mid-treatment, immediate and long-term outcomes will be measured. Based on prior research, we hypothesise that the therapist-guided form will be superior to the unguided form in reducing anxiety levels, and that both delivery modes will be superior to an active waiting-list control group.

## Methods/Design

### Design

This randomised controlled trial has been registered in the clinicaltrials.gov registry (NCT01963806) and has received approval from the Region Ethical Review Board in Stockholm (approval nr: 2013/880-31/5).

**Figure 1:**
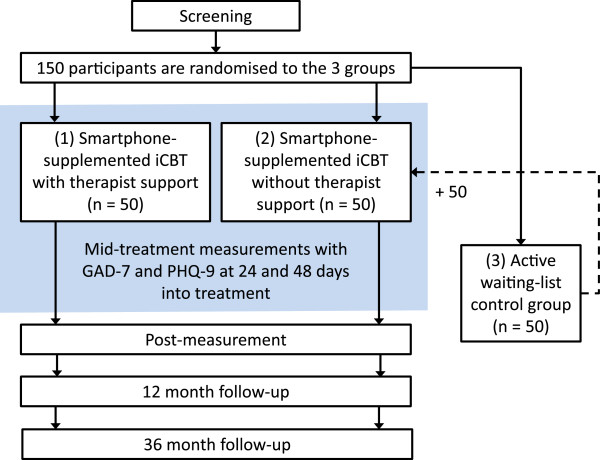
Study flowchart.

Upon inclusion, participants will be randomised by an independent researcher using an online randomisation tool to one of three study groups: 1, iCBT supplemented with a smartphone application and therapist support; 2, iCBT supplemented with a smartphone application without therapist support; or 3, an active waiting-list control group. As in most psychotherapeutic interventions, blinding participants to study arm allocation is not possible in this case. After the two treatment groups have completed the post measurements, the control group will receive the same treatment as group 2 for ethical reasons. This also entails that there will be no control group to compare with at the follow-up measurements at 12 and 36 months after treatment. See Figure 1 for study flowchart.

### Procedure

The recruitment and screening procedure will be similar to other recent iCBT studies by our research group [22,23]. The study will be advertised nationwide in press and online. Prospective participants will be directed to a public website (http://www.actsmart.se) where they will find further information on the study and what participation entails. Screening is done online via a dedicated platform and includes questions on demographics and treatment history, as well as the self-rating scales that serve as outcome measures: the 7-item Generalised Anxiety Disorder (GAD-7) [24], the Liebowitz Social Anxiety Scale (LSAS-SR) [25,26], Panic Disorder Severity Scale (PDSS-SR) [27,28], the 9-item Patient Health Questionnaire (PHQ-9) [29] and the Quality of Life Inventory (QOLI) [30]. Diagnostic interviews using the Structured Clinical Interview for DSM disorders (research edition) [31] will be conducted per telephone [32].

The individual pretreatment data collected from the screening will be reviewed by the research team, led by an experienced clinical psychologist and psychotherapist. Participants meeting inclusion criteria will be randomised to study arms and either begin treatment (groups 1 and 2) or be informed that they will receive treatment after a 10-week delay (group 3).

### Sample

The study sample will consist of adults (>18 years old) living in Sweden with sufficient Swedish and daily access to the Internet by both computer and smartphone. Participants must satisfy DSM-IV-TR [33] diagnostic criteria for either social anxiety disorder (SAD) or panic disorder (PD), or both. Primary diagnosis will be determined during the clinical interview, and will be used as a between-group factor in second-stage analyses. Participants enrolled in a parallel psychological treatment will not be included. Ongoing, regular psychoactive medication will not serve as grounds for exclusion if dosage has been stable during the last 3 months. Sporadic use of psychoactive medication (for example, beta blockers) will be allowed. Should suspicion arise at any point prior to commencing treatment or during the treatment period that a participant is suffering from other treatment-requiring disorders (including psychiatric ones), the participant will be encouraged to seek local care.

Participants who do not meet inclusion criteria will be personally encouraged to seek treatment alternatives better suited to their specific needs. Included participants will be given a unique, anonymous participant number with which to log in to the dedicated, SSL-encrypted online platform. Single-use login codes sent by SMS to their registered telephone number will be used to further guarantee secure login. All included participants will be asked to provide written informed consent before commencing treatment. Participants who drop out during the treatment period will not be replaced.

### Interventions

#### Treatment modules

All participants will receive the same self-help program, either with or without therapist support. The core of the treatment program has been developed and empirically tested by our research team in numerous studies stretching over a decade [10]. Previous clinical RCTs using the same core treatment modules have demonstrated post-treatment, between-group Cohen’s d effect sizes in the range of d = 0.79^a^[34] and 0.98^b^[35] for SAD and d = 1.44^c^[36] and 1.97^d^[37] for PD when contrasted against waiting-list control groups.

In this trial, the treatment program will be divided into eight modules covering CBT and Acceptance and Commitment Therapy (ACT) conceptualisations of anxiety disorders, as well as more specific therapeutic techniques such as cognitive restructuring, exposure training, acceptance training, goal-setting according to values, attention and breathing exercises, and relapse prevention. All treatment modules will be accessible from the online platform at once, allowing individual pacing [38].

#### Smartphone application

**Figure 2:**
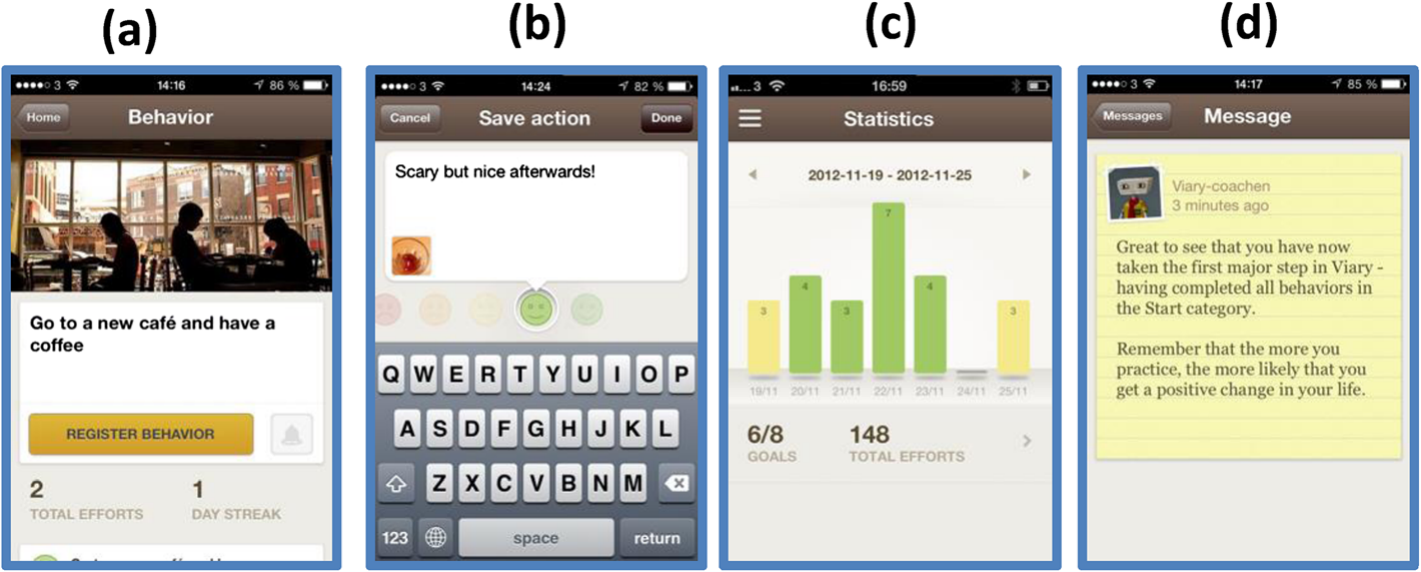
**Screenshots of application in simulated use. (a)** Choosing a behaviour from repertoire. **(b)** Saving a comment after carrying out a behaviour. **(c)** Statistics of carried-out behaviours. **(d)** Feedback.

In addition to these modules, the treatment program will also include a smartphone application tailored for this specific study. The application is integrated with the exercises from the treatment modules, which will encourage participants to make frequent use of the application throughout the program. The core features of the application used in this study have been described elsewhere [39] and are designed to facilitate behaviour change. Briefly, the purpose of the application is to help the user remember and keep track of key behaviours, in order to promote everyday activation. When a behaviour is completed, the user can register this using the application and also add a short reflection. Encouraging, automated feedback is then given. Statistics and summaries of quantitative (that is, behaviour frequencies) and qualitative data (that is, reflections) can be accessed by both the user and an assigned therapist. The therapist can also send short text messages to the participants via an in-built messaging system. In this study, this messaging system will be used by the therapists to send tailored, encouraging messages to the participants in study group 1, approximately two or three times a week. Participants will not be able to reply to these messages. As a rule of thumb, the therapists will devote 15 minutes per participant and week. Participants in study group 2 will only receive the automated feedback from the application (Figure 2).

### Instruments

With the exception of the clinical interview conducted at screening via telephone, all measurements will be collected via the dedicated online platform. Previous psychometric research has validated Internet-administration of self-rating scales for social anxiety, panic attacks, quality of life, and general depression and anxiety [40-42]. Established Swedish translations of all instruments will be used. Measurements will be collected from all participants at screening, immediately and 12 and 36 months after the treatment period. Additionally, measurements with the GAD-7 and PHQ-9 only will be completed 24 and 48 days into treatment in order to detect rapid treatment effects. All post-treatment measurements will include questions on potential adverse outcomes ascribable to treatment, as well as on significant changes in life situation (for example, divorce) and changes in parallel treatment status (for example, change in medication dosage or having commenced a parallel psychological treatment).

#### Primary outcome

The Generalised Anxiety Disorder 7-item (GAD-7) scale will serve as a transdiagnostic measure of common anxiety symptoms and will constitute the primary outcome measure. Although designed to measure symptoms corresponding to the DSM-IV diagnosis of generalised anxiety disorder (GAD), the symptoms assessed by the GAD-7 should be considered common across all anxiety disorders (for example, excessive worrying, trouble relaxing and so on). The seven items of the scales are rated 0 to 3 (‘Not at all’ to ‘Nearly every day’) based on their occurrence within the last 2 weeks. Further psychometric evaluation has reported good internal consistency, factor structure and sensitivity to change following treatment [43].

#### Secondary outcomes

As this study will include participant with SAD and/or PD, diagnosis-specific self-rating scales will complement measurements of generic anxiety symptoms. Participants will complete the self-rated Liebowitz Social Anxiety Scale (LSAS-SR) [25,26] and Panic Disorder Severity Scale (PDSS) [27,28]. Since we expect some co-morbidity between these two disorders, all participants will answer both instruments. All participants will also answer the 9-item Patient Health Questionnaire (PHQ-9) [29,44,45] to measure depressive symptoms, as well as the Quality of Life Inventory (QOLI) [30,46] to provide measure of symptom-independent quality of life.

### Calculations and analyses

This study aims to detect a moderate-sized, post-treatment between-group difference (Cohen’s d = 0.5) with 80% power, which will require 150 participants distributed evenly to the three groups. In the post-hoc analyses contrasting any two groups, there will be 80% power to detect a d = 0.66 effect size when Bonferroni-adjusting the *P* values for the three possible post-hoc comparisons. When comparing each treatment group with the control group, we hypothesise effect sizes larger than d = 0.66 based on previously studies reporting higher effect sizes for the same core treatment program compared to a waiting-list control group [34-37].

Since all self-reported data are collected using the online platform, there is no risk of missing data, or loss or distortion of data. All analyses will be conducted on an intention-to-treat basis using a mixed models approach [47]. Power calculations were performed using the pwr package (http://CRAN.R-project.org/package=pwr) for the *R* statistical environment (http://www.r-project.org/; version 2.15.3).

## Discussion

Supplementing iCBT with tailored smartphone applications is a promising way to expand the reach of this already effective intervention. This study protocols describes a RCT designed to answer two questions important for the future use of smartphone-supplemented iCBT: is smartphone-complemented iCBT an effective treatment of social anxiety disorder and panic disorder; and, will the addition of support from a therapist impact results? Few studies so far have investigated the effects of smartphone-supplemented iCBT interventions on mental health, and no study has examined the effect on anxiety disorders specifically.

This study is not designed to compare regular iCBT-only *versus* smartphone-supplemented iCBT and hence does not feature a study arm receiving the former treatment alternative. Due to similarities in recruitment procedure and the core intervention components (that is, the treatment modules), we will be able to make cautious comparisons with the effect sizes found in previous research on regular iCBT-only by our research group. However, future studies featuring a 2 × 2 intervention design (iCBT treatment modules yes/no × smartphone application yes/no) will be necessary to disentangle the specific effect of each component used in the current study. Another limitation to the current study design is that the control group, for ethical reasons, will receive treatment in the second stage of the trial, entailing that there will be no control group during the follow-up period when long-term effects are studied. Further, blinding participants to study arm allocation is not feasible in this study.

These limitations notwithstanding, the results of this trial will constitute important advancements in the burgeoning field of mobile health interventions, specifically smartphone-supplemented iCBT interventions. If found to be effective, off-the-shelf available, customisable smartphone applications could with negligible effort and costs be integrated into both iCBT self-help programs and traditional face-to-face CBT, benefiting both patients and healthcare providers.

## Trial status

At time of initial submission, this trial was recruiting. Recruitment was opened in September 2013 and closed in October 2013 when 150 participants had been included.

## Endnotes

^a^aEffect size calculated on the LSAS-SR scores.

^b^bEffect size calculated on the LSAS-SR fear/avoidance scores.

^c^Effect size calculated on the Body Sensation Questionnaire scores.

^d^Effect size calculated on the Body Sensation Questionnaire scores.

## Abbreviations

ACT: Acceptance and commitment therapy; CBT: Cognitive behavioural therapy; DSM-IV-TR: Diagnostic and statistical manual of mental disorders fourth edition (Text-revised); GAD-7: Generalized anxiety disorder 7-item; iCBT: Internet-administered cognitive behavioural therapy; LSAS-SR: Self-rated Liebowitz Social Anxiety Scale; PDSS: Panic disorder severity scale; PHQ-9: Patient health questionnaire 9-item; SCID: Structured clinical interview for DSM disorders.

## Competing interests

Author KHL is developing a related version of the smartphone application intended for the open market and will thus not be involved in data analysis or any decisions related to the publication of findings. The other authors declare that they have no competing interests.

## Authors’ contributions

PL drafted the manuscript and facilitated the conception of the study. EI is co-responsible for the execution of the study, coordinates efforts and serves as therapist and clinical interviewer. KHL developed and is responsible for the smartphone application featured in this study. GA and PC designed the study and facilitated its conception. PC serves as principal investigator. All authors participated in the review and revision of the manuscript and have approved the final version for publication.
